# Sodium-Glucose Cotransporter-2 Inhibitor-Induced Euglycemic Diabetic Ketoacidosis Followed by Excessively Low Carbohydrate Diet

**DOI:** 10.7759/cureus.16085

**Published:** 2021-07-01

**Authors:** Erika Tsutsui, Yoji Hoshina, Hirofumi Homma

**Affiliations:** 1 Department of Diabetes and Metabolic Diseases, The University of Tokyo Hospital, Tokyo, JPN; 2 General Medicine, Chiba University Hospital, Chiba, JPN

**Keywords:** sodium-glucose cotransporter2 inhibitor, euglycemic diabetic ketoacidosis, type 2 diabetes mellitus, low carbohydrate diet, lifestyle modification

## Abstract

Euglycemic diabetic ketoacidosis (euDKA) is a rare but serious adverse effect caused by sodium-glucose cotransporter-2 (SGLT2) inhibitors, and it can be challenging to identify in the emergency room (ER). In this report, we present a case of a type 2 diabetic patient whose diagnosis was delayed due to the absence of marked hyperglycemia. A 39-year-old female presented to the ER with a four-day history of nausea, vomiting, sweating, dyspnea, and generalized weakness after initiating dapagliflozin, along with a low carbohydrate diet and moderate exercise to curb her newly diagnosed type 2 diabetes mellitus (DM). However, this had resulted in strict avoidance of carbohydrates. Despite seeking medical attention twice, a proper diagnosis had been delayed due to the absence of marked hyperglycemia. Her blood glucose level at our hospital was 181 mg/dl and urine analysis showed ketonuria and glucosuria. She was admitted to the ICU with a diagnosis of euDKA related to SGLT2 inhibitor use. She was successfully treated with insulin and glucose supplementation.

## Introduction

Diabetic ketoacidosis (DKA) is a serious and potentially life-threatening acute complication of diabetes mellitus (DM). It is characterized by the triad of hyperglycemia (blood glucose level >250 mg/dl), ketosis, and metabolic acidosis (arterial pH <7.3 and serum bicarbonate <18 mEq/L). In rare cases, patients can present with blood glucose levels of less than 200 mg/dl, which is defined as euglycemic DKA (euDKA) [1,2]. It is challenging to identify euDKA in the emergency room (ER) due to the absence of marked hyperglycemia, often leading to delayed diagnosis and treatment. DKA and euDKA have been recently found to be associated with sodium-glucose cotransporter-2 (SGLT2) inhibitors, one of the newest classes of oral hypoglycemic agents [1,2]. We describe the case of a type 2 diabetic patient with euDKA secondary to dapagliflozin initiation along with strict avoidance of carbohydrates and moderate exercise, which could have been prevented with thorough patient education.

## Case presentation

A 39-year-old Asian female presented to our hospital with nausea, vomiting, sweating, dyspnea, and generalized weakness. She had been diagnosed with type 2 DM six days prior by her primary care physician (PCP), with elevated HbA1c (13.0%) and hyperglycemia (300 mg/dl). She had been advised to start a low carbohydrate diet and daily exercise and had been started on dapagliflozin (5 mg). However, this had resulted in strict avoidance of carbohydrates on her part. Two days after the initiation of dapagliflozin, she had developed nausea, vomiting, and loss of appetite. Due to severe fatigue, she had been evaluated both in the local ER and by her PCP who had assured her that her blood glucose level was unremarkable, and her symptoms might be due to hyperventilation and dehydration. However, her symptoms had continued to worsen, and she came to our ER the following day.

The patient had no fever, chills, abdominal pain, alcohol intake history, nor any history of recent surgery. On physical examination, the patient was drowsy, but alert and oriented to time, place, and person. Her vital signs were as follows: blood pressure of 108/83 mmHg, heart rate of 114 beats/minute, respiratory rate of 20 breaths/minute, and temperature within normal limits. Oxygen saturation was 98% on room air. Her height was 155 cm, and her weight was 63 kg (body mass index of 26.2 kg/m^2^). Oral mucosa was dry but otherwise unremarkable. Initial laboratory data showed significant metabolic acidosis with a blood pH of 7.19, bicarbonate level of 10.4 mmol/L, and anion gap of 25.0 mmol/L without an elevation in lactate. Her blood glucose was 181 mg/dl and her HbA1c was 12.6%. Urinalysis showed 4+ ketones and glucose >1000 mg/dl. The anti-insulin antibody was negative. Other laboratory data on admission are shown in Table 1. She was admitted to the ICU with a diagnosis of euDKA related to SGLT2 inhibitor use. Her glucose levels were monitored on an hourly basis, and her basic metabolic panel and venous blood gas were monitored until the gap was closed. Seven hours after admission, the anion gap was closed. On day two of admission, she was able to start having meals and her laboratory abnormalities were found rectified. We provided diabetic education including home blood sugar monitoring and discharged the patient with insulin glargine 10 units every evening and glucose tablets in case of hypoglycemia. The patient was followed up by a local diabetologist. Insulin was discontinued with the initiation of metformin a week after the discharge. Anti-glutamic acid decarboxylase (GAD) antibody was investigated by the specialist and was found negative. Her DM has been well controlled since her discharge without any recurrent episodes of ketoacidosis.

**Table 1:** Laboratory data on admission WBC: white blood cells; RBC: red blood cells; Hb: hemoglobin; Plt: platelet; T-bil: total bilirubin; AST: aspartate aminotransferase; ALT: alanine aminotransferase; ALP: alkaline phosphatase; TP: total protein; Alb: albumin; UN: urea nitrogen; Cr: creatinine

Hematology		Metabolic panel			
WBC	10.4 x 10^3^/mcl	T-bil	0.8 mg/dl	K	4.8 mmol/l
RBC	5.35 x 10^6^/mcl	AST	15 U/l	Cl	108 mmol/l
Hb	13.4 g/dl	ALT	15 U/l	HCO_3_-	10 mmol/l
Plt	50.5 x 10^3^/mcl	ALP	127 U/l	Glucose	181 mg/dl
Blood gas analysis (venous)		TP	9.0 g/dl	Lactate	1.0 mmol/l
pH	7.19	Alb	5.4 g/dl	Urinalysis	
pCO_2_	27.4 mmHg	UN	13 mg/dl	Glucose	>1000 mg/dl
pO_2_	40 mmHg	Cre	0.6 mg/dl	Ketone	4+
HCO_3_-	10.4 mmol/l	Na	139 mmol/l		

## Discussion

We discussed a case of euDKA following the initiation of dapagliflozin with an excessively low carbohydrate diet. SGLT2 inhibitors are a Food and Drug Administration (FDA)-approved medication widely used in patients with type 2 DM and in limited form in patients with type 1 DM [3]. Recent studies have associated the medication with favorable outcomes in reducing hospitalization for heart failure and progression of renal disease [3]. On the other hand, diabetic ketoacidosis has been reported as one of the adverse effects of SGLT2 inhibitors, apart from acute kidney injury, dehydration, and hypotension. In May 2015, the FDA issued a warning that SGLT2 inhibitors may lead to ketoacidosis [4]. The known contributing factors include reduction or omission of insulin, severe acute illness, dehydration, extreme physical activity, surgery, low caloric and fluid intake, or excessive alcohol intake [5]. These factors can cause relative or absolute insulin deficiency that leads to reduced glucose use and disinhibition of glucagon release from alpha cells in the pancreas. This stimulates lipolysis in adipose tissue and enhanced ketogenesis in the liver, which increases ketones in the blood [1,2]. SGLT2 inhibitors may also inhibit glucose transportation to alpha cells through SGLT and promote the further release of glucagon, which causes further free fatty acid oxidation and ketosis. Additionally, SGLT2 may promote renal tubular reabsorption of acetoacetate [6]. This impaired glucose utilization leads to ketosis without overt hyperglycemia since SGLT2 inhibitors block glucose reabsorption in the renal proximal tubule [1,2].

This case is notable for two key reasons. Although it is difficult to find a direct association, the patient’s euDKA may have been precipitated by a low carbohydrate diet, which is normally advised in type 2 DM patients [7]. The patient continued to have an excessively low carbohydrate diet due to a lack of awareness about its adverse effects, which could have been prevented with thorough patient education. Furthermore, the patient did not have a clear understanding of what a "low" carbohydrate diet entailed. In fact, ketoacidosis triggered by "no-carbohydrate" diets has been previously reported [8]. Although a low carbohydrate diet is not a leading precipitating factor of SGLT2 inhibitor-induced euDKA, this case illustrates the importance of proper patient education, as lifestyle modification is routinely advised for diabetic patients. Secondly, this case shows that the time of onset of SGLT2 inhibitor-induced euDKA can be very short. In our patient, symptoms started only two days after SGLT2 inhibitor initiation. According to FDA data, the median time of onset of SGLT2 inhibitor-induced DKA is 43 days [4]. The exact mechanism behind this variable time in onset is not well understood, but in this case, it may have been caused by sudden excessive changes in lifestyle, which could have been prevented with better patient education.

Lastly, while euDKA from SGLT2 inhibitors is a well-documented side effect, cases are still frequently overlooked, leading to delayed diagnosis and patient treatment. This illustrates the need for continued provider education and raising awareness about this condition, along with the fact that the presentation timeframe is highly variable.

## Conclusions

Patients with type 2 DM are normally advised to have a low carbohydrate diet and perform moderate exercise. This case highlights the importance of imparting appropriate patient education, given that these lifestyle modifications can potentially lead to the development of euDKA, especially when SGLT2 inhibitors are included in their regimen. Patients on SGLT2 inhibitors should be informed that there is a risk of developing euDKA even if it is monotherapy and the patients have no history of severe illness nor any history of recent surgery.

Furthermore, this case illustrates the challenge in diagnosing euDKA in the ER due to the absence of marked hyperglycemia; euDKA can develop within a very short period after the initiation of SGLT2 inhibitor treatment. Hence, euDKA should be considered in patients with DKA symptoms who are on SGLT2 inhibitor medication so that prompt measures can be initiated, especially since this is a life-threatening condition.
